# Accuracy of echocardiographic estimations of right heart pressures in adult heart transplant recipients

**DOI:** 10.1002/clc.23835

**Published:** 2022-04-22

**Authors:** Aadhavi Sridharan, Monica M. Dehn, Craig Cooper, Vidya S. Madineedi, Linda J. Ordway, David DeNofrio, Ayan R. Patel

**Affiliations:** ^1^ Cardiovascular Imaging and Hemodynamic Laboratory Tufts Medical Center Boston Massachusetts USA; ^2^ Heart Failure and Cardiac Transplant Program Tufts Medical Center Boston Massachusetts USA

**Keywords:** echocardiography, heart transplant recipients, pulmonary artery systolic pressure, right atrial pressure

## Abstract

**Background:**

Accurate assessment of right atrial pressure (RAP) and pulmonary artery systolic pressure (PASP) is critical in the management of heart transplant recipients. The accuracy of echocardiography in estimating these pressures has been debated.

**Objective:**

To assess the correlation and agreement between echocardiographic estimations of right heart pressures with those of respective invasive hemodynamic measurements by right heart catheterization (RHC) in adult heart transplant recipients.

**Methods:**

This is a prospective evaluation of 84 unique measurements from heart transplant recipients who underwent RHC followed by standard echocardiographic evaluation within 159 ± 64 min with no intervening medication changes. The relationship between noninvasive pressure estimations and invasive hemodynamic measurements was examined.

**Results:**

Mean RAP was 7 ± 5 mmHg and mean PASP was 33 ± 8 mmHg by RHC. There was no significant correlation between echocardiographic estimation of RAP and invasive RAP (Spearman's rho = −0.05, *p* = .7), and no significant agreement between these two variables (weighted kappa = −0.1). There was a modest correlation between echocardiographic estimation of PASP and invasive PASP (*r* = .39, *p* = .002). Bland‐Altman analysis showed a mean bias of 2.1 ± 9 mmHg (limits of agreement = −15 to 20 mmHg).

**Conclusion:**

In heart transplant recipients, there is no significant correlation or agreement between echocardiographic RAP estimation and invasively determined RAP. Noninvasive PASP estimation correlates significantly but modestly with invasively measured PASP. Further refinement of echocardiographic methods for assessment of RAP is warranted in this unique patient population.

**Abbreviations**
ASE: American Society of Echocardiography
IVC: inferior vena cava
PASP: pulmonary artery systolic pressure
RAP: right atrial pressure
RHC: right heart catheterization
TR: tricuspid regurgitation
TTE: transthoracic echocardiogram

## 1 INTRODUCTION

Right atrial and pulmonary artery systolic pressures (RAP, PASP) have significant clinical implications in the diagnosis, management, and prognosis of cardiac and pulmonary disorders.^1^, ^2^, ^3^, ^4^, ^5^ While invasive right heart catheterization (RHC) remains the gold standard method for the systematic measurement of these pressures, its use is limited due to the invasiveness of the procedure and potential risk of complications such as infection, thrombosis, arrhythmias, pulmonary artery rupture, and right ventricular perforation.^6^ Thus, there is an obvious clinical need for noninvasive methods of estimating these pressures, and echocardiography is currently widely used in this clinical context. However, the accuracy of these echocardiographic estimations especially in special patient populations such as adult heart transplant recipients has not been previously studied. The objective of the current study was to assess the correlation and agreement between echocardiographic estimations of RAP and PASP with that of respective invasive hemodynamic measurements by RHC in a prospective, blinded study design.

## 2 METHODS

Adult heart transplant recipients who were referred for RHC and transthoracic echocardiogram (TTE) on the same day at Tufts Medical Center as part of their standard clinical treatment plan were identified and approached for participation in the study. Patients were enrolled in the study from June 2018 to May 2019. Exclusion criteria included mechanical ventilation, pulmonic stenosis, concurrent left heart catheterization, or inability to provide informed consent. All patients provided written informed consent before enrollment in the study. This study was approved by the Tufts Medical Center Institutional Review Board.

Patients underwent RHC via jugular or femoral venous access. RAP, right ventricular systolic and diastolic pressures, PASP and pulmonary artery diastolic pressure, and pulmonary capillary wedge pressure were obtained, with zero‐reference level set at the midthoracic level.^7^ Hemodynamic values were obtained at end‐expiration by an observer who was blinded to the echocardiographic data. Cardiac output and index were measured using the Fick and/or thermodilution methods. Patients undergoing concurrent left heart catheterization after RHC were excluded from the study to avoid any confounding effect of contrast use on hemodynamic values.

All patients then underwent TTE following RHC, to avoid any confounding effects of moderate sedation on hemodynamic values. All structural and functional chamber assessments were made according to the American Society of Echocardiography (ASE) guidelines.^8^ Specifically, the inferior vena cava (IVC) diameter was measured by 2D echocardiography, perpendicular to the long‐axis of the IVC in the subcostal view, with the patient in the supine position, 1–2 cm from the junction of the IVC and right atrium. The percent IVC collapse with normal respiration was calculated as the difference of maximum diameter and minimum diameter divided by the maximum diameter. The upper limit of normal IVC diameter was defined as 2.1 cm and >50% IVC collapsibility index was considered normal. IVC diameter below or equal to 2.1 cm and IVC collapsibility index above 50% were considered consistent with a RAP of 3 mmHg. IVC diameter >2.1 cm and IVC collapsibility index below 50% were considered to be consistent with a RAP of 15 mmHg. Measurements not conforming to either of these combinations were estimated to have a RAP of 8 mmHg. PASP was estimated using the modified Bernoulli equation from the peak tricuspid regurgitant (TR) jet velocity and adding the estimated RAP. TR flow was identified using color Doppler, and peak TR jet velocity was measured by aligning continuous wave spectral Doppler signal as parallel to the direction of TR flow as possible, per ASE guidelines.^9^ The single observer interpreting the echocardiographic data was blinded to the hemodynamic values obtained by RHC. Patients with poor echocardiographic windows limiting accurate IVC diameter and IVC collapsibility index measurements, and/or insufficient TR jet were excluded from study analysis.

Continuous variables are expressed as mean ± standard deviation, and categorical variables as proportions. Spearman's rho or Pearson correlation coefficient was used to evaluate the association between echocardiographic estimations and invasive hemodynamic measurements by RHC, and weighted kappa and Bland‐Altman analysis^10^, ^11^ were used to assess the agreement between echocardiographic estimations and invasive hemodynamic measurements.

## 3 RESULTS

A total of 84 serial hemodynamic and echocardiographic evaluations in heart transplant recipients were included in the data analysis. Patients underwent TTE 159 ± 64 min after RHC with no intervening medication changes. Patients were 54 ± 11 years of age at the time of evaluation, and were 193 ± 294 days from cardiac transplantation (range: 4–2593 days). Forty‐eight (57%) of the patients were male. Additional basic demographic, anthropometric, hemodynamic, and echocardiographic data are provided in Table 1.

**Table 1:** Patient characteristics (*n* = 84)

Variable	
Age (years)	54 ± 11
BMI (kg/m^2^)	29 ± 5
Male	48 (57%)
Time from heart transplant (days)	193 ± 294
LVEF on TTE (%)	57 ± 5
Moderate or severe TR (%)	5 (6%)
Time lag between RHC and TTE (minutes)	159 ± 64
Systolic blood pressure (mmHg)	146 ± 15
Diastolic blood pressure (mmHg)	94 ± 13
Heart rate (BPM)	97 ± 15
Right atrial pressure (mmHg)	7 ± 5
Pulmonary capillary wedge pressure (mmHg)	14 ± 7
Pulmonary artery systolic pressure (mmHg)	33 ± 8
Pulmonary artery diastolic pressure (mmHg)	16 ± 6
Fick cardiac output (L/min)	6 ± 1.2
Fick cardiac index (L/min/m^2^)	3 ± 0.6

*Note*: Continuous variables are reported as mean ± standard deviation, and categorical variables as *n* (%). Abbreviations: BMI, body mass index; BPM, beats per minute; LVEF, left ventricular ejection fraction; RHC, right heart catheterization; TR, tricuspid regurgitation; TTE, transthoracic echocardiogram.

**Figure 1:**
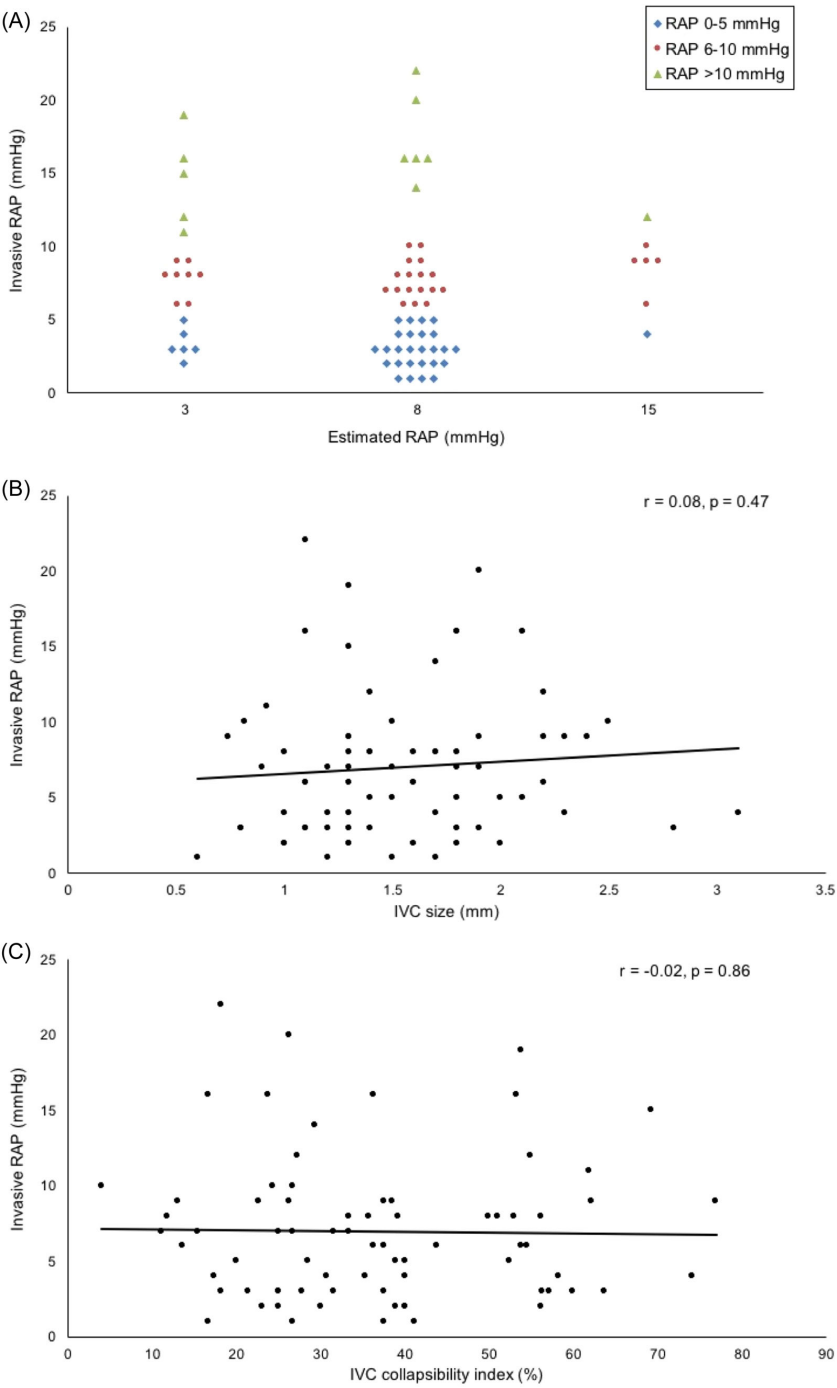
Relationship between noninvasively estimated and invasively measured right atrial pressures. (A) Beeswarm plot of estimated right atrial pressure (RAP) by echocardiography and invasively measured RAP. (B) Association between inferior vena cava (IVC) diameter and invasively measured RAP. (C) Association between IVC collapsibility index and invasively measured RAP. RAP indicates right atrial pressure and IVC indicates inferior vena cava

IVC assessment was not feasible in 10 patients due to suboptimal echocardiographic windows and poor visualization of the IVC. Mean RAP was 7 ± 5 mmHg (range: 1–22 mmHg) by RHC. There was no significant correlation between echocardiographic estimation of RAP and invasive RAP (Spearman's rho = −0.05, *p* = .7; Figure 1A), and there was no significant agreement between these two variables (weighted kappa = −0.1). Similarly, there was no significant correlation between IVC diameter (*r* = .08, *p* = .47, Figure 1B) or IVC collapsibility index (*r* = −0.02, *p* = .86, Figure 1C).

Sensitivity of echocardiographic estimation of normal RAP (0–5 mmHg) was 0.18 (95% CI: 0.7–0.32), specificity of echocardiographic estimation of high RAP (>5 mmHg) was 0.59 (95% CI: 0.41–0.76), and accuracy of echocardiographic estimation of RAP was 0.38 (95% CI: 0.27–0.51).

**Figure 2:**
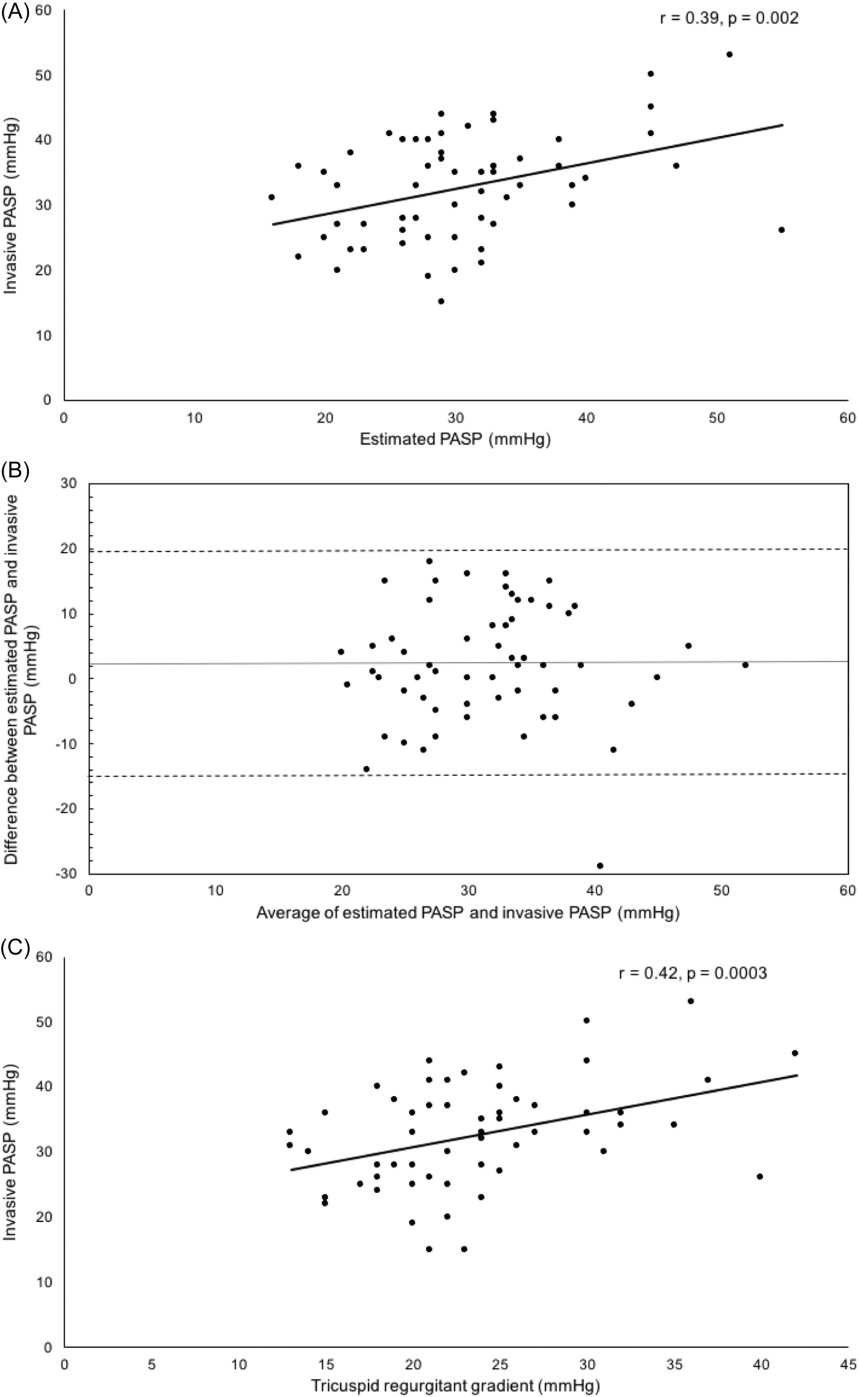
Relationship between noninvasively estimated and invasively measured pulmonary artery systolic pressure (PASP). (A) Association between estimated PASP by echocardiography and invasively measured PASP. (B) Bland‐Altman plot of estimated PASP and invasively measured PASP. Dotted line represents mean bias of 2.1 mmHg, and dashed lines represent limits of agreement (–15 to 20 mm Hg). (C) Association between tricuspid regurgitant gradient and invasively measured PASP. PASP indicates pulmonary artery systolic pressure

Accurate PASP estimation was not feasible in 16 patients due to lack of sufficient TR jet. Mean PASP was 33 ± 8 mmHg (range: 15–63 mmHg) by RHC. There was a modest correlation between echocardiographic estimation of PASP and invasive PASP (*r* = .39, *p* = .002; Figure 2A). Bland‐Altman analysis showed a mean bias of 2.1 ± 9 mmHg (limits of agreement = −15 to 20 mmHg; Figure 2B). There was also a modest but significant correlation between TR gradient measured by Doppler echocardiography and invasive PASP (*r* = .4223, *p* = .0003, Figure 2C).

## 4 DISCUSSION

This is the first study to evaluate the association and agreement between echocardiographic estimations of RAP and PASP compared to respective hemodynamic measurements in adult heart transplant recipients. We show that noninvasive echocardiographic assessments do not accurately estimate RAP and estimate PASP only with modest accuracy in this unique patient population. That there is only a modest (but significant) association between TR gradient measured by Doppler echocardiography and invasively measured PASP may possibly be due to suboptimal alignment of the continuous wave Doppler beam with TR flow to obtain maximum TR jet velocity. Further, the lack of significant correlation between estimated RAP by echocardiography and invasively measured RAP suggests that the addition of estimated RAP to the modified Bernoulli equation to obtain an estimation of the PASP only amplifies the error in estimating PASP using echocardiography.

Our finding that echocardiographic estimation of RAP does not accurately predict actual RAP is perhaps not surprising given multiple conflicting prior reports.^12^ Natori et al. first described that IVC lumen decreased during inspiration, reached a minimum diameter at the end of inspiration, distended again during expiration, and closed transiently 2–3 cm below the diaphragm during maximal inspiration.^13^ Since then, while several studies have evaluated the correlation between RAP and various IVC parameters, far fewer studies have critically assessed the validity of the current guideline‐recommended IVC indices in accurately estimating RAP.^12^ Tsutsui et al. observed that echocardiographic RAP prediction methods showed only modest precision and wide limits of agreement with invasive measurements in patients with acutely decompensated advanced heart failure.^14^ Most recently, Magnino et al. tested six different IVC‐based schemes of RAP estimation and demonstrated that RAP estimation based on the IVC is highly inaccurate regardless of the method used, subsequently concluding that this should be avoided whenever possible.^15^

The inaccuracy of echocardiography in estimating RAP specifically in heart transplant recipients may also be related to the currently practiced surgical technique of heart transplantation. In orthotopic heart transplantation with bicaval and left atrial anastomoses, there is total excision of the recipient's right atrium, and the donor's heart implantation is performed using bicaval end‐to‐end anastomoses as well as left atrial anastomosis.^16^ Whether such mechanical disruption of the IVC due to caval anastomosis alters the compliance of the vessel and the normal respirophasic change in IVC size and dynamics, thereby affecting its correlation to RAP has not been previously described, and warrants further research.

Our study findings have important clinical implications. Extensive prior evidence demonstrates the diagnostic and prognostic value of RAP and PASP in diverse patient populations, thus accurate measurement of these parameters is crucial in these clinical settings. RAP provides important information about a patient's overall volume status. Elevated central venous pressure is associated with poor outcomes and prolonged treatment in critical care settings, and is correlated with impaired renal function and all‐cause mortality in patients with a wide spectrum of cardiovascular diseases.^2^, ^5^ Persistently elevated right‐sided filling pressure in patients with heart failure during a heart failure hospitalization is predictive of the combined risk of death, cardiovascular hospitalization, and heart transplantation.^17^ Similarly, in patients with hypertrophic cardiomyopathy, elevated RAP is associated with left‐sided heart failure and is an independent predictor of all‐cause mortality and new‐onset atrial fibrillation.^18^ In patients with pulmonary hypertension, elevated PASP is independently associated with increased mortality, and elevated RAP (suggestive of right ventricular diastolic dysfunction) is closely associated with poor outcomes.^19^ Echocardiography is widely used to infer a diagnosis of pulmonary hypertension as well as to longitudinally follow the effects of treatment in patients with pulmonary hypertension. Registry data in this patient population shows that changes in mean RAP and mean pulmonary artery pressure predict survival.^20^ However, similar to our current finding that echocardiography is only modestly accurate in estimating PASP in heart transplant recipients, several prior reports have documented that Doppler echocardiography frequently inaccurately estimates PASP in patients with pulmonary hypertension.^21^, ^22^, ^23^ Given the inaccuracies of RAP estimation and the amplification of measurement errors by using derived variables, current guidelines recommend using the continuous wave Doppler measurement of peak TR velocity, and not the estimated PASP which includes estimated RAP, as the main variable in determining the echocardiographic probability of pulmonary hypertension.^4^, ^24^

Timely and accurate assessment of RAP and PASP is especially critical in heart transplant recipients. Right ventricular dysfunction remains prevalent in heart transplant recipients, may require the use of mechanical circulatory support, and if unrecognized in a timely manner may result in fatal outcomes.^25^, ^26^ The pathogenesis of right ventricular dysfunction following heart transplantation is likely multifactorial, and may be due to acute graft dysfunction, pulmonary hypertension, insufficient preservation causing myocardial ischemia/reperfusion injury, arrhythmias, inflammatory mediators resulting from brain death, or less commonly due to pulmonary artery stenosis at the anastomosis site.^25^ RAP has been shown to be associated with primary graft failure and risk of mortality in heart transplant recipients.^27^, ^28^ Elevated pulmonary artery pressure at first annual evaluation after heart transplantation predicts poor outcomes in these patients.^29^ Given the findings of the current study, therefore, the clinical use of echocardiography to noninvasively estimate intracardiac right heart pressures specifically in this specific population may be unreliable. While current ASE guidelines for evaluating left ventricular diastolic function clearly indicate that assessment of diastolic function is more difficult in heart transplantation using standard methods,^30^ no such specification is made in the guidelines related to estimation of right heart pressures.^8^ Further refinement of echocardiographic methods for assessment of RAP is needed in this unique patient population.

There are several limitations to the present study. First, the current study only included a modest number of patients from a single heart transplant center. Second, while we tried to minimize the time lag between RHC and TTE, noninvasive estimation of intracardiac pressures were not done simultaneously at the time of invasive hemodynamic measurements due to logistical constraints and to optimize image quality during TTE. However, no intervening medication changes were made in the time between the right heart invasive hemodynamic measurements and the echocardiogram, thereby lessening the likelihood of major changes in hemodynamics during that time interval. Additionally, we used the minimum end‐inspiratory IVC diameter during spontaneous respiration rather than a sniff maneuver. The analysis was performed with this approach to preserve the image acquisition plane, to minimize any error in IVC size measurement due to movement, as well as to avoid variation in technique between patients, some of whom may not be able to perform a sniff maneuver.

In conclusion, echocardiography does not accurately estimate RAP and only modestly estimates PASP in adult heart transplant recipients. Further research is needed in identifying accurate noninvasive indicators of RAP in this special patient population.

## Data Availability

The data that support the findings of this study are available from the corresponding author upon reasonable request.
